# Microradiography as a useful technique for the rapid detection of skeletal anomalies in early sea bream juveniles

**DOI:** 10.1111/jfd.13622

**Published:** 2022-04-09

**Authors:** Chrysovalentinos Pousis, Mariasevera Di Comite, Rosa Zupa, Letizia Passantino, Edmond Hala, Aldo Corriero

**Affiliations:** ^1^ Department of Emergency and Organ Transplantation Section of Veterinary Clinics and Animal Production University of Bari Aldo Moro Valenzano Italy; ^2^ Department of Basic Medical Sciences Neurosciences and Sensory Organs University of Bari Aldo Moro Bari Italy; ^3^ Department of Animal Production Faculty of Agriculture and Environment Agricultural University of Tirana Kamez, Tirana Albania

**Keywords:** juvenile fish, microradiography, sekeletal anomalies

## 1 INTRODUCTION

As for any industrial sector, the aquaculture industry needs to minimize production costs to increase business profit and competitiveness. A source of profit loss for finfish hatcheries is the one related to skeletal anomalies and the resulting fish deformities. Farming conditions can induce morphofunctional anomalies and changes in fish physiology and behaviour (Almeida et al., 2008; Corriero et al., 2011; Kihslinger & Nevitt, 2006; Zupa et al., 2013, 2017). Skeletal anomalies may cause negative effects on fish growth, welfare and health (Koumoundouros et al., 2001; Sfakianakis et al., 2006), thus increasing mortality rates and decreasing product marketability (Boglione, Gavaia, et al., 2013). The financial loss related to skeletal anomalies for Mediterranean hatcheries has been estimated to exceed 50 million € per year (Boglione, Gisbert, et al., 2013).

Unfavourable rearing conditions (Fernández et al., 2008; Izquierdo et al., 2010), genetic factors (Fragkoulis et al., 2018; Negrín‐Báez, Navarro, Lee‐Montero, et al., 2015), nutrition and other factors (Boglione, Gavaia, et al., 2013; Boglione, Gisbert, et al., 2013) have been shown to induce skeletal anomalies in various cultured fish species. Skeletal anomalies originate as a slight aberration of the internal fish anatomy during the embryonic, larval and early juvenile stages (Boglione et al., 2009; Fragkoulis et al., 2017) and subsequently they can develop into more severe abnormalities affecting the external body shape (Boglione, Gisbert, et al., 2013; Divanach et al., 1997; Koumoundouros et al., 2001). The skeletal anomalies with the highest incidence in hatchery‐produced sea bream and sea bass affect the head (Divanach et al., 1996) and the vertebral column (Negrín‐Báez et al., 2015).

Deformed fishes are often downgraded to filets or fish meal as they are not suitable to be sold as whole fish on the market (Boglione et al., 2003, 2009; Hilomen‐Garcia, 1997; Lijalad & Powell, 2009). Moreover, severely deformed fish can induce consumers to lose confidence in aquaculture products (Boglione, Gisbert, et al., 2013).

The early identification of skeletal anomalies is economically advantageous because (i) hatchery managers may decide to discard a fish lot showing a high incidence of anomalies and (ii) a high incidence of anomalies may represent an indicator of non‐conformities in the production process. Moreover, an early diagnosis and follow‐up of skeletal anomalies may provide useful information regarding the possible recovery during fish growth. A recent study (Fragkoulis et al., 2019) showed that some skeletal anomalies might spontaneously regress during fish growth.

Diagnosis of skeletal anomalies in teleost fish during the first developmental stages is usually obtained on samples processed through an in toto double staining (DS) in which cartilage and bone are stained with alcian blue and alizarin red, respectively, and non‐skeletal tissues are made translucent by a clearing agent (Hanken & Wassersug, 1981; Park & Kim, 1984; Wassersug, 1976). This method provides good anatomic detail, and it can be simultaneously applied to groups of animals. However, this method is not suitable for large fish because it would require a long processing time and a high amount of reagents; hence fish with a total length >60 mm are analysed by radiography (Boglione et al., 2001). This method generates a negative image of fish skeleton, and it is also applicable to alive fish, provided that suitable X‐ray equipment (X‐ray generator and detector) is available in the farming facility. However, the common radiological technique is not suitable for studying skeletal anomalies at early developmental stages because low bone density produces low‐contrast and low‐resolution images (Boglione et al., 2001). To overcome these problems, different procedures using soft X‐rays (low energy and long‐wavelength X‐rays) have been proposed (Bonhan & Bayliff, 1953; Hjelde & Bæverfjord, 2009; Miller & Tucker, 1979). Microradiography is a high‐resolution, fine detail, contact X‐rays technique used for microscopic examination of histological sections and evaluation of bone mineral density and distribution (Sterchi, 2013). This technique requires a powerful, controllable source of “soft” X‐rays (low kV) and high‐resolution film or photographic plates. Samples must be in tight, close contact with the film or plate and developed under controlled conditions for comparative work (Sterchi, 2013).

This study aimed to set up a protocol to process early sea bream juveniles for microradiography and assess possible advantages and limits of the use of microradiography for the identification of skeletal anomalies in early juveniles of a commercially reared fish species through the comparison with the commonly used DS method.

## 2 MATERIALS AND METHODS

### 2.1 Sampling

A total of 92 juveniles of gilthead sea bream, ranging from 18 to 31 mm total length (TL), reared in an Italian commercial hatchery were sampled by hand net and transferred to a beaker where they were anaesthetized with a lethal dose of anaesthetic (0.4 ml/L of 2‐phenoxyethanol, Merck) and fixed in 10% formalin buffered with phosphate buffer (pH 7.2) for 48 h at 4°C. Fixed samples were then washed in phosphate‐buffered saline (pH 7.5) for 48 h at 4°C and then stored in 70% ethanol until further processing.

### 2.2 Microradiography and double in toto staining

All the samples were hydrated in 50% ethanol for 24 h, divided into groups of four to seven specimens of the same length (difference in TL within each group <1 mm). All the fish of each group were then micro‐radiographed together in latero‐lateral projection according to the method described by Amprino and Engstrom (1952). Contact microradiographs were obtained on Kodak high‐resolution film (SO 343, Eastman Kodak Co., Rochester, NY, USA) using an X‐ray generator (Constant 1‐K, Ital Structures, Italy). The fish were placed on a dedicated specimen holder at a prefixed distance of 9.5 cm from the X‐ray generator. Since the high‐resolution film is sensitive to water, a transparent polyethylene film was placed between the photographic film and the specimens. X‐ray exposure was set up at 12 kV and 18 mA. The specimens <25 mm TL were subjected to 6500 X‐ray shots, while the specimens ≥25 mm TL underwent 7500 shots. Films were then developed with Kodak HC‐110, fixed in Kodak UNIFIX, washed in distilled water and then dried at room temperature.

After microradiography, all the samples were stored overnight in 50% ethanol before being processed for DS according to the procedure reported by Park and Kim (1984) and modified by Cataudella et al. (2014) and by the present authors to get the following staining protocol. The samples were (i) immersed in a solution of 0.5% KOH and 3% H_2_O_2_ and exposed to sunlight for 3–4 h; (ii) incubated in 100 ml cartilage staining solution (60 ml 95% ethanol, 40 ml acetic acid, 25 mg alcian blue) for 90 min in the dark at room temperature; (iii) washed three times for 10 s in 50 ml of 0.5% KOH; (iv) stained in 100 ml 0.03% alizarin red solution (30 mg alizarin red in 100 ml of 0.5% KOH) for 1 h in the dark at room temperature; (v) washed three times for 10 s in 50 ml of 0.5% KOH and (vi) diaphanized by consecutive immersions in solutions containing 0.5% KOH and increasing glycerol concentrations (0.5% KOH:glycerol 3:1; 0.5% KOH:glycerol 1:1; 0.5% KOH:glycerol 1:3; 100% glycerol). The duration of the first two steps in the clearing solutions was about 48 h, while the third step was repeated several times until the 0.5% KOH:glycerol 1:3 solution remained colorless after 24 h of immersion.

### 2.3 Stereoscopic analysis

Microradiographic plates, as well as DS specimens, were observed under a stereomicroscope (Leica WILD M3C, Wetzlar, DE), and all the fish were photographed by a digital camera (DFC 420; Leica Microsystems, Cambridge, UK). To compare the sensitivity of the two methods, each specimen was assigned an identification code, its meristic characters were counted, and the skeletal anomalies were identified and recorded. At the end of the microscopic analysis, all the specimens were individually stored in vials previously filled with glycerol. To reduce possible errors during the analysis of the samples, the specimens were observed three times at different times by the same operator. The recorded meristic characters were: vertebrae (including the urostyle); fin‐supporting elements (epural, hypural, pterygiophores and radial); rays of dorsal, anal, caudal and pectoral fins.

The anatomical terminology used in the present study complies with the one adopted by Prestinicola et al. (2013), which is based on definitions provided by Harder (1975), Matsuoka (1987) and Schultze and Arratia (1989). According to Prestinicola et al. (2013), skeletal anomalies were classified as mild or severe: severe anomalies are those affecting the external shape of the fish body (i.e., lordosis, kyphosis, vertebral fusions and jaw anomalies), whereas mild anomalies are those that do not alter the external morphology.

## 3 RESULTS AND DISCUSSION

**FIGURE 1:**
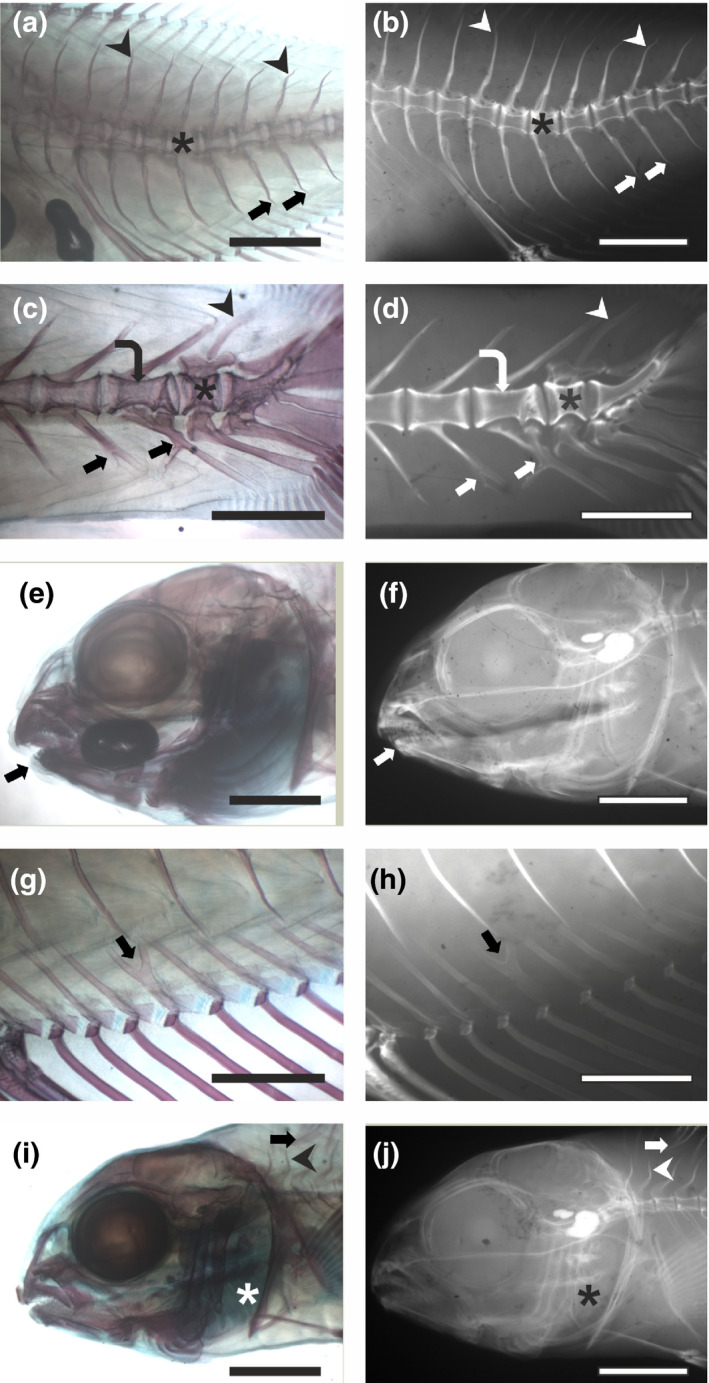
Skeletal anomalies in sea bream juveniles processed with a double in toto staining (left) and microradiography (right). (a‐b) Haemal lordosis (asterisk) between the 12th and 18th vertebra; several anomalous neural (arrowheads) and haemal (arrows) spines are also visible. (c‐d) Partial fusion of two caudal vertebrae (curved arrow); the deformed body of a caudal vertebra (asterisk) and anomalous neural (arrowhead) and haemal (arrows) spines are also present. (e‐f) Anomalous dentary (lower jaw reduction). (g‐h) Anomalous (forked) anal pterygiophore (arrow). (i‐j) Anomalous opercular plate; anomalous neural spine (arrowhead) and pterygiophore (arrow) are also visible Magnification bars: 2 mm in (a), (b), (e), (f), (i) and (j) and 1 mm in (c), (d), (g) and (h)

A total of 248 skeletal anomalies were observed by both methods in the exanimated specimens; the majority of the analysed individuals (82.6%) showed at least one skeletal anomaly and most of them showed more than one anomaly (average anomalies per individual: 3.3 ± 2.4). The most frequent observed anomalies wereanomalies affecting caudal vertebrae bodies (19.6% of the specimens); anomalies affecting neural and haemal arches of caudal vertebrae (25% of the specimens) and anomalies affecting the caudal fin as part or total fused epurals and hypurals (43.5% of the specimens). Among the analysed specimens, 50.0% were affected by severe anomalies, such as haemal lordosis (V‐shaped curvature in the vertebral column; Figure 1a, b); vertebral body shape anomalies, marked reduction in length or elongation of the vertebral body, vertebral body fusion (Figure 1c‐d) and cephalic deformities (Figure 1e, f).

Thirty‐four specimens (37.0% of the analysed specimens) showed mild anomalies without co‐occurrence of severe anomalies (Figure 1g, h). The following mild anomalies were observed: anomalous neural or haemal spines; supernumerary bones; anomalous hypurals and epurals and anomalous pterygiophores.

The presence of urinary calculi was observed in 34% of the examined specimens by both methods. This pathology does not involve skeletal elements; however, it is often considered in studies on fish skeletal anomalies because it represents a severe and potentially lethal condition (Boglione et al., 2001).

Incidentally, the types of observed anomalies and the respective incidences are in agreement with the literature data on the skeletal anomalies recorded in hatchery‐produced sea bream fingerlings (Boglione et al., 2001; Prestinicola et al., 2013).

Samples processed with both methods displayed fair good anatomical details. Although the internal fin‐supporting elements (radials, hypurals and epurals), as well as fin rays of some specimens, were weakly marked in the microradiographic plates due to the lower mineral content, all the anomalies of these elements observed in DS samples were correctly recorded also in microradiographic plates. Therefore, the main limitations of the use of microradiography for the detection of skeletal anomalies in early sea bream juveniles are represented by the low resolution obtained for some fin‐supporting elements and, in some cases, a difficult identification of anomalies affecting only one of paired skeletal elements (Figure 1i‐j). Although the double staining method provides better details of some internal elements and allows easier identification of anomalies affecting paired structures, its execution requires several days of laboratory work, thus delaying the availability of the diagnosis. The use of microradiography requires a very limited time from sampling to diagnosis because this method can be applied directly to fish killed with a lethal dose of anaesthetic and then dehydrated in 50% ethanol for 30–120 min.

Therefore, microradiography, despite a slightly lower sensitivity compared to the in toto staining, provided a clear and timely picture of the incidence of skeletal anomalies in sea bream fingerlings with TL ranging between 18 and 31 mm, thus representing a useful tool for the rapid identification of production non‐conformities in a phase of rapid fish growth. Further studies are required to determine the size range of applicability of this method.

## CONFLICT OF INTEREST

The authors declare that there is no conflict of interest.

## Data Availability

The data that support the findings of this study are available from the corresponding author upon reasonable request.
